# Uncovering the Cellular and Molecular Landscape of Gynecological Disorders

**DOI:** 10.3390/cells14171399

**Published:** 2025-09-08

**Authors:** Qiwei Yang

**Affiliations:** Department of Obstetrics and Gynecology, University of Chicago, Chicago, IL 60637, USA; yangq@bsd.uchicago.edu

## 1. Introduction

Gynecological disorders encompass a diverse array of conditions that affect the female reproductive system, including the uterus, ovaries, fallopian tubes, cervix, vagina, and external genitalia. These disorders range from common benign conditions, such as uterine fibroids, endometriosis, and polycystic ovary syndrome (PCOS), to malignancies, including ovarian, cervical, uterine leiomyosarcoma, and endometrial cancers. Collectively, they impose a significant burden on women’s physical, reproductive, and psychological health, often leading to chronic pain, infertility, and impaired quality of life [1,2,3,4]. Importantly, gynecological cancers remain a leading cause of cancer-related morbidity and mortality among women worldwide, with late-stage diagnoses and limited treatment options contributing to poor long-term outcomes in many cases [5,6]. Beyond the individual impact, both benign and malignant gynecological diseases contribute to substantial societal and economic costs through healthcare expenditures, loss of productivity, and long-term disability [7,8]. In recent years, breakthroughs in molecular biology, genomics, epigenomics, and advanced imaging technologies have profoundly expanded our understanding of the cellular and molecular underpinnings of these conditions. These advances have uncovered both distinct and overlapping mechanisms driving the pathogenesis of benign and malignant gynecological diseases, suggesting shared regulatory networks that span the spectrum of reproductive pathology [9,10]. For example, intricate crosstalk between the hormonal and immune systems has emerged as a central feature influencing tissue homeostasis, inflammation, and disease progression. Likewise, the role of cellular senescence in shaping the microenvironment [11], as well as dynamic remodeling of the extracellular matrix (ECM) [12,13], is now recognized as a key determinant of disease onset and chronicity.

Moreover, emerging evidence implicates autophagy and inflammasome activation as important modulators of cellular stress responses and immune activation in gynecologic tissues. These insights are reshaping our conceptual frameworks for how gynecological disorders originate and evolve. At the same time, innovative technologies such as single-cell RNA sequencing (scRNA-seq), epigenetic-based omics analysis, spatial transcriptomics, and patient-derived organoid models are revolutionizing the study of reproductive diseases. These platforms enable high-resolution dissection of cellular heterogeneity, allow for more refined disease classification, and accelerate the discovery of novel biomarkers and therapeutic targets. Together, these developments are transforming gynecological disease research, paving the way toward more precise, mechanism-based diagnostic and therapeutic strategies.

This collection of reviews and original research articles offers a comprehensive and timely overview of emerging insights into the cellular and molecular mechanisms underlying ovarian and uterine pathologies. The included studies span a diverse yet interconnected spectrum of gynecologic conditions, highlighting the growing recognition of shared pathogenic processes across both benign and malignant diseases. Topics covered include mesothelial clearance by endometriosis spheroids, impaired decidualization, autophagy in reproductive aging, protease regulation in ovarian function, immune–tumor interactions in ovarian cancer, and the molecular drivers of fibrosis, senescence, transformation, and tumorigenesis in uterine tissues. Collectively, these contributions underscore the urgent need for integrated research approaches to advance novel diagnostics, fertility-preserving interventions, and precision therapies for gynecologic diseases.

## 2. An Overview of Published Articles

### 2.1. Female Fertility and Cellular Mechanisms

Two articles in this Special Issue focus on gynecological disorders that affect multiple organs, including both the ovaries and the uterus. Autophagy, a highly conserved cellular process vital for maintaining intracellular homeostasis, plays a pivotal role in female reproductive function [14]. Recent studies underscore the essential role of autophagy in embryo implantation, particularly within the endometrium, where it supports optimal fetal development. However, despite these advances, critical gaps remain in our understanding of how autophagy affects oocyte quality, follicular development, and overall reproductive capacity at the molecular level. Autophagy contributes to oocyte maturation and developmental competence and is implicated in reproductive aging by influencing age-related declines in fertility. The review article by Harrath et al. (contribution 1) explores the physiological roles of autophagy across the female reproductive system and its involvement in reproductive toxicity. The authors further summarize the functional links between autophagy, endometrial receptivity, and embryo development. Moreover, this review article highlights the intricate relationship between autophagy and various aspects of female reproductive health, including pregnancy, ovarian function, infertility, endometriosis, and gynecologic malignancies. Emerging therapeutic strategies aimed at modulating autophagy through both natural compounds and synthetic agents are also discussed, with the goal of enhancing female fertility and improving reproductive outcomes. By synthesizing current findings and identifying existing knowledge gaps, this review aims to stimulate further research into the multifaceted roles of autophagy in reproductive biology. A comprehensive understanding of autophagy’s impact on female fertility holds promise for advancing diagnostics, therapeutics, and the broader field of reproductive medicine.

Oxidative stress plays a key role in accelerating the age-related decline of reproductive function and fertility [15]. Recent research has identified human amniotic membrane-derived mesenchymal stem cells (AMSCs) as a promising tool for reducing oxidative damage and promoting tissue repair. In an original study by Ra (contribution 2), intravenous administration of AMSC-conditioned medium (AMSC-CM) was shown to reduce oxidative stress and improve reproductive function in aged female mice. The treatment modulated reproductive hormone levels to resemble those of younger mice and induced beneficial gene expression changes in the ovaries and uterus, supporting anti-aging, energy metabolism, and reproductive functions. These results suggest that AMSC-CM holds therapeutic potential for combating age-related female infertility through its antioxidative and regenerative properties.

The ovary plays a central role in gynecological disorders, including polycystic ovary syndrome (PCOS), ovarian cysts, endometriosis, and ovarian cancer [16,17], all of which can disrupt hormone balance, fertility, and reproductive health. Proteases are key regulators of ovarian folliculogenesis, guiding processes from primordial follicle activation to ovulation and corpus luteum formation. These enzymes mediate essential cellular and tissue-level functions such as extracellular matrix (ECM) remodeling, cell proliferation, apoptosis, and intercellular signaling—each vital for proper follicular development and ovarian function [18]. The review article by Kushawaha and Pelosi (contribution 3) provides a comprehensive synthesis of current knowledge on the classification, regulation, and function of proteases in ovarian physiology and pathology. The authors highlight several major protease families, including matrix metalloproteinases (MMPs), tissue inhibitors of metalloproteinases (TIMPs), the plasminogen activator (PA) system, and cathepsins, discussing their specific contributions to follicle development. These proteases tightly regulate ECM turnover and cellular communication, facilitating the dynamic structural changes required for follicular growth, oocyte maturation, and luteal phase transition. Beyond normal ovarian physiology, dysregulated protease activity has been implicated in several ovarian pathologies. The authors explore the involvement of aberrant proteolytic signaling in the development and progression of ovarian cancer, polycystic ovary syndrome (PCOS), and primary ovarian insufficiency (POI), highlighting how altered expression or activity of specific proteases contributes to disease mechanisms. Finally, by integrating findings from clinical genomic studies and mechanistic investigations using animal models, the authors underscore the translational potential of targeting proteases for therapeutic intervention. This review provides a foundation for future research into protease-based diagnostics and treatments in reproductive medicine and ovarian disease.

Chronic inflammation is increasingly recognized as a key contributor to female reproductive dysfunction [16], affecting both natural conception and the success of assisted reproductive technologies such as in vitro fertilization (IVF). As a central mediator of innate immune responses, the NLR family pyrin domain-containing 3 (NLRP3) inflammasome plays a pivotal role in inflammatory signaling and is closely associated with oxidative stress. Activation of the NLRP3 inflammasome in response to oxidative stress has been linked to reduced oocyte quality, compromised fertilization potential, and impaired embryo development. Within the ovarian microenvironment, the interplay between oxidative stress and NLRP3 activation exerts a substantial impact on oocyte competence and reproductive outcomes. The review article by Moustakli et al. (contribution 4) elucidates the molecular mechanisms underlying NLRP3 inflammasome activation and its interaction with oxidative stress in the context of female fertility. Additionally, the authors explore emerging therapeutic strategies that target these pathways to enhance oocyte quality, support embryo development, and ultimately improve reproductive success.

### 2.2. Ovarian Cancer: Molecular Pathways and Biomarkers

Hypoxia-Inducible Factor-1α (HIF-1α) plays a pivotal role in the progression of multiple cancers by promoting cellular adaptation, survival, angiogenesis, metabolic reprogramming, immune evasion, and metastasis [19]. The review article by Rahman et al. (contribution 5) elucidates the molecular functions of HIF-1α in ovarian cancer and infertility, highlighting innovative therapeutic strategies aimed at both disease control and fertility preservation. Under hypoxic conditions, HIF-1α translocates to the nucleus, where it dimerizes with HIF-1β and binds to hypoxia-response elements (HREs) in the promoter regions of target genes. This activation drives the expression of genes involved in angiogenesis, metabolic reprogramming, epithelial-to-mesenchymal transition, therapeutic resistance, and immune evasion. HIF-1α–mediated angiogenesis and glycolytic reprogramming support tumor proliferation, survival, and metastasis. Concurrently, dysregulation of HIF-1α disrupts ovarian homeostasis by impairing follicular development, hormone production, and vascular integrity, processes essential for female fertility. Additionally, HIF-1α promotes chronic inflammation and oxidative stress, further compromising reproductive health. Given its dual role in tumor progression and infertility, HIF-1α is an attractive therapeutic target. Emerging strategies, including small-molecule inhibitors and nanoparticle-based drug delivery systems, hold promise for reducing HIF-1α activity, thereby limiting cancer progression while safeguarding reproductive function.

Epithelial ovarian cancer (EOC) is the most common and aggressive form of ovarian cancer and represents the leading cause of gynecological cancer-related deaths in women. It is often diagnosed at an advanced stage, contributing to its poor prognosis. Unlike many other cancers, EOC spreads through a unique metastatic mechanism involving the formation of multicellular spheroids within the peritoneal cavity [20]. The study by Tomas et al. (contribution 6) offers valuable insights into the signaling plasticity driving this process, using an EOC spheroid model. The authors propose that EOC cells undergo biological shifts between tumor and spheroid states during cancer dormancy. By directly comparing cultured EOC spheroids and organoids, they found increased AMPK activity and decreased Akt signaling in spheroids, indicating distinct phenotypes. RNA sequencing further revealed key differences, particularly in cell cycle regulation in organoids. The inhibition of the G2/M checkpoint confirmed its essential role in organoid viability. Notably, both spheroids and organoids showed equal sensitivity to Alisertib, highlighting AURKA as a potential therapeutic target. These findings underscore the need to understand EOC cell adaptations, as therapeutic vulnerabilities may vary across disease stages.

In addition to investigating signaling plasticity in ovarian cancer using two 3D models by Tomas (contribution 6), Kang et al. (contribution 7) identified prognostic biomarkers for high-grade serous carcinoma (HGSC), a highly lethal malignancy characterized by initial chemotherapy sensitivity and frequent recurrence. Unlike prior studies limited to bulk RNA sequencing or a few genetic markers, this study utilized spatial transcriptomics and multispectral immune cell immunofluorescence (IF) to uncover markers associated with disease progression post-first-line treatment. Notable findings included elevated NKG7 expression in CD45^+^ immune regions and increased TFPI2 and PIGR in tumor areas, all linked to improved progression-free survival. Multispectral IF further revealed higher Treg/CD8^+^ T cell ratios and closer proximity of Tregs to cancer cells and macrophages in recurrent tumors—correlating with poor outcomes. Integrated analyses demonstrated that immune cell density and immune pathway scores in recurrent tumors were positively correlated with cancer pathway activity, excluding NF-κB. These results offer new insights into immune–tumor interactions and potential biomarkers for predicting HGSC recurrence.

### 2.3. Uterine Aging, Fibrosis, and Genetic Mutations

The uterus is the central organ linked to the highest prevalence and diversity of gynecological conditions. Fibrosis in the uterus often arises from chronic inflammation, repeated injury, hormonal dysregulation—particularly involving estrogen and progesterone—or aberrant wound-healing responses. Characterized by excessive accumulation of extracellular matrix (ECM) proteins, uterine fibrosis disrupts normal reproductive function and contributes to infertility in humans. The review article by Matsuno and Imakawa (contribution 8) explores the role of uterine fibrosis and biological aging as underrecognized yet potentially critical contributors to declining fecundity in cattle, which serve as valuable research models. The authors detail the mechanisms of fibrosis in the female reproductive tract of cattle, highlighting key pathways such as TGF-β, β-catenin, and Hippo signaling, as well as hormonal interactions that form a complex cross-regulatory network amplifying fibrotic responses. Additionally, the review addresses the impact of biological aging—including molecular drivers, epigenetic aging, and cellular senescence—on uterine function and infertility. Targeting fibrotic signaling pathways may offer promising avenues for the development of non-surgical therapies for uterine fibrotic disorders.

Uterine fibroids, or leiomyomas (UFs), are often associated with excessive extracellular matrix (ECM) deposition and fibrotic remodeling, contributing to their firm, fibrous nature and disease progression [13]. UFs are common benign tumors affecting up to 70–80% of women by age 50. Arising from the myometrium, they cause symptoms like abnormal bleeding, pelvic pain, infertility, and pregnancy complications, and are a major reason for hysterectomy. Genomic studies have identified *MED12* gene mutations—especially in exon 2—as the most frequent alteration, found in 60–70% of UFs. These mutations, which disrupt mediator-associated CDK activity, transcription, and chromatin regulation [21,22], likely drive the clonal expansion of myometrial stem cells but are rarely seen in malignant uterine leiomyosarcomas (ULMS), suggesting a benign trajectory. The cause of *MED12* mutations remains unclear. The article by Li et al. (contribution 9) demonstrated that *MED12* mutations can be detected in myometrial cells from hysterectomy specimens using duplex deep sequencing (DDS), which offers higher accuracy and lower error rates compared to standard next-generation sequencing (NGS) methods. The authors found that oxidative stress promotes *MED12* mutations in myometrial cells and is associated with increased DNA damage, suggesting that it may be a significant risk factor for the acquisition of these mutations. This study provides important insights into the molecular basis underlying the high incidence of uterine fibroids in women of reproductive age.

The endometrium is essential for reproductive success, facilitating implantation and pregnancy through tightly regulated processes involving hormonal responsiveness, immune modulation, and tissue regeneration. However, aging disrupts these functions, with cellular senescence—a state of irreversible cell cycle arrest induced by DNA damage and oxidative stress—emerging as a key contributor. The review article by Kobayashi et al. (contribution 10) summarizes the molecular mechanisms underlying cellular senescence in age-related endometrial dysfunction. Although senescence plays beneficial roles in tumor suppression and tissue repair, its dysregulation can impair endometrial integrity and compromise fertility. Central to the regulation of senescence are signaling pathways involving p53, AMPK, and mTOR, which coordinate cellular stress responses, metabolic regulation, and proliferation control. Specifically, p53 activates AMPK and inhibits mTOR, thereby promoting energy conservation and limiting excessive senescence. AMPK further suppresses mTOR activity, mitigating age-associated cellular dysfunction. This p53–AMPK–mTOR axis, together with autophagy, plays a pivotal role in determining cell fate in response to stress and nutrient availability. While moderate levels of senescence may support endometrial function, excessive accumulation of senescent cells can disrupt tissue homeostasis and reduce fertility. A deeper understanding of these molecular interactions may guide the development of targeted strategies to counteract reproductive aging and improve fertility outcomes.

### 2.4. Endometriosis: Immune and Molecular Pathophysiology

Endometriosis is a gynecologic disorder characterized by the presence of endometrium-like glandular and stromal tissue outside the uterine cavity. The role of hormonal dysregulation, particularly estrogen, is well established in the initiation, progression, and persistence of the disease [23]. Emerging evidence also underscores the involvement of immune system dysfunction in the pathophysiology of endometriosis. The human endometrium is a highly dynamic tissue that undergoes cyclical remodeling driven by hormonal cues throughout the menstrual cycle. Similarly, ectopic endometrial lesions exhibit hormone-responsive behavior, though their pathogenic mechanisms remain incompletely understood. This complexity poses significant challenges to fully elucidating the etiology and pathology of the disease. The review article by Greygoose et al. (contribution 11) explores the intricate interplay between estrogen and both the innate and adaptive immune systems during the menstrual cycle, focusing on their roles in the development and progression of endometriosis. Estrogen has been shown to modulate numerous inflammatory and immunoregulatory processes, influencing the behavior of both tissue-resident and circulating immune cells. The authors highlight key estrogen-mediated interactions with specific myeloid and lymphoid cell populations, emphasizing their contributions to the immunopathogenesis of endometriosis. To investigate the pathology and lesion spread in endometriosis, Kloeckner and Walker (contribution 12) developed an in vitro mesothelial clearance assay using spheroids of endometriotic and normal epithelial cells cultured on a mesothelial monolayer. They found that both cell types could mediate mesothelial clearance, with endometriotic spheroids showing enhanced clearance ability. This assay offers a useful platform for testing potential therapies, though further research is needed to understand the mechanisms of mesothelial clearance and whether endometriotic lesions can invade the underlying extracellular matrix, similar to ovarian cancer.

Defective decidualization has been mechanistically linked to uterine conditions that impair successful conception, such as endometriosis [24]. However, the presence and underlying causes of decidualization defects in the eutopic endometrium of women with endometriosis remain largely unclear. In this review, Retis-Resendiz et al. (contribution 13) integrate and critically evaluate molecular evidence from both in vivo and in vitro studies that have investigated alterations in decidualization within the eutopic endometrium of endometriosis patients. Multiple studies have reported impaired decidualization, implicating dysregulation in key genes such as *IGFBP1*, *PRL*, *connexin 43*, *HOXA10*, and *FOXO1*. Disruptions have also been observed in several signaling pathways, including sex steroid hormone signaling via genomic and non-genomic mechanisms, TGF-β signaling through SMAD2/3/4, bone morphogenetic protein signaling through SMAD1/5/4, and Notch signaling. Additionally, pro-inflammatory cytokines and epigenetic mechanisms, such as DNA methylation and histone modifications, have been shown to contribute to the dysregulation of the decidualization process. Despite these insights, further functional studies are needed to determine whether these molecular defects directly contribute to endometriosis-associated infertility. A deeper understanding of the decidualization process and its disruption in endometriosis may not only inform fertility-targeted therapies but also support the development of more effective treatments for this complex and chronic condition.

Increasing evidence implicates immune system dysregulation, particularly innate immunity and Toll-like receptors (TLRs), in the development and progression of diseases [25]. The study by Sobstyl et al. (contribution 14) investigated the expression of specific TLRs (TLR2, TLR3, TLR4, TLR7, TLR8, and TLR9) on peripheral blood lymphocyte subpopulations (CD4^+^, CD8^+^, and CD19^+^ cells) in women with endometriosis. The authors also measured the levels of their soluble forms in serum and urine. The results revealed significantly increased TLR expression in lymphocyte subpopulations of endometriosis patients compared to healthy controls, indicating active involvement of TLR pathways in both systemic and localized immune responses. Receiver operating characteristic analysis further demonstrated the potential of TLR profiles to distinguish endometriosis patients from controls and possibly differentiate among disease subtypes. These findings reinforce the role of TLRs in the immunopathogenesis of endometriosis and highlight their promise as diagnostic biomarkers and therapeutic targets. Further studies with larger cohorts and mechanistic investigations of TLR signaling are warranted to confirm and expand upon these observations.

### 2.5. Decoding Benign and Malignant Uterine Pathologies

Recent advances in transcriptomics, especially single-cell RNA sequencing (scRNA-seq), have transformed our understanding of uterine biology by revealing the cellular diversity and molecular mechanisms driving these conditions [26]. The review article by Boldu-Fernandez et al. (contribution 15) summarizes current research on mapping the cellular architecture of the several gynecological diseases through scRNA-seq technology. In the endometrium and myometrium, scRNA-seq and spatial transcriptomics uncovered key cell types, signaling pathways, and dynamic changes across the menstrual cycle. These technologies have also highlighted functional abnormalities in stromal and immune cells, such as fibroblast-to-myofibroblast transitions and impaired macrophage function, that contribute to fibrosis, chronic inflammation, and lesion persistence in endometriosis. In endometrial cancer, scRNA-seq has revealed the complexity of the microenvironment, identifying distinct cancer-associated fibroblast subtypes and immune cell profiles that influence disease progression and therapy resistance. Similarly, studies of adenomyosis have identified disrupted signaling pathways, including Wnt and VEGF, and novel progenitor cell populations associated with tissue invasion and neuroinflammation. In UFs, single-cell analyses have characterized smooth muscle and fibroblast subpopulations involved in extracellular matrix remodeling and key pathways such as ERK and mTOR. While challenges like scalability and reproducibility remain, the authors concluded that multi-omics high-resolution single-cell analyses hold great promise for biomarker discovery, therapeutic target identification, and the development of personalized treatments for gynecological diseases.

ULMS is a rare but highly aggressive malignancy arising from the smooth muscle cells of the myometrium. Representing approximately 1–2% of all uterine cancers, ULMS is clinically and biologically distinct from the far more common benign UFs. ULMS is characterized by rapid growth, high recurrence rates, and poor prognosis, with a five-year survival rate of less than 40% in advanced stages [9]. Emerging evidence highlights the role of epigenetic dysregulation in ULMS [27,28,29,30,31]. However, the contribution of BET (bromodomain and extra-terminal domain) proteins to ULMS pathogenesis has not been fully elucidated. In this study by Yang et al. (contribution 16), members of the BET protein family, including BRD2, BRD3, and BRD4, were found to be aberrantly overexpressed in ULMS tissues compared to normal myometrium. Pharmacological inhibition of BET proteins using small-molecule inhibitors JQ1 and I-BET762 significantly suppressed ULMS cell proliferation in a dose-dependent manner by inducing cell cycle arrest. Notably, high-throughput RNA-sequencing analysis revealed that targeted inhibition of BET proteins led to alterations in several critical signaling pathways, including the Hedgehog pathway, epithelial–mesenchymal transition (EMT), and transcription factor-driven regulatory networks. In addition, BET inhibition impacted multiple epigenetic regulators, including DNA methyltransferases, histone-modifying enzymes, and N6-methyladenosine RNA methylation machinery, suggesting broader epigenetic remodeling because of BET targeting. These findings not only highlight BET proteins as potential biomarkers of disease progression but also suggest that BET inhibition could serve as a promising therapeutic strategy for targeting dysregulated transcriptional and epigenetic programs in ULMS.

## 3. Future Perspectives

As our understanding of gynecological disorders continues to deepen, future research must leverage emerging cellular and molecular insights to address the persistent clinical challenges these conditions present. The integration of advanced omics technologies, high-resolution imaging, and patient-derived models offers unprecedented opportunities to dissect disease complexity at single-nucleotide, single-cell, and spatial resolution. These cutting-edge approaches will be pivotal in redefining disease taxonomies, shifting from traditional histopathological classifications toward molecularly informed subtypes that more accurately capture the underlying biology and therapeutic vulnerabilities.

A critical next step in gynecological disease research is the integration of multi-omic datasets, including genomic, transcriptomic, epigenomic, proteomic, and metabolomic information, to build comprehensive molecular atlases of gynecologic tissues across developmental stages, physiological states, and disease progression. These integrative frameworks will be essential for uncovering early pathogenic drivers, identifying dynamic biomarkers of disease onset and progression, and revealing novel therapeutic targets. In parallel, the expanded application of patient-derived organoids, advanced ex vivo systems, and in vivo imaging technologies will enhance the translational pipeline, enabling more predictive and physiologically relevant preclinical testing, particularly for understudied or aggressive malignancies such as uterine leiomyosarcoma.

Clinical translation research should focus on developing diagnostic approaches grounded in underlying disease mechanisms, such as non-invasive biomarkers and risk stratification assays, augmented by Artificial Intelligence (AI) technologies to enable earlier detection and personalized management of gynecological diseases [32,33]. Efforts to develop low-cost, high-throughput platforms and to ensure the inclusion of diverse populations in biomarker discovery studies are also crucial. Leveraging AI-driven analytics alongside these strategies will be essential to translating scientific advances into accessible, equitable solutions that improve early detection, guide treatment decisions, and ultimately enhance health outcomes for all women globally.

## 4. Concluding Remarks

This Special Issue presents a diverse, multidisciplinary collection of original research and review articles that advance our understanding of the molecular, cellular, and physiological mechanisms underlying a broad spectrum of gynecological disorders. The contributions address ovarian and uterine diseases, infertility, endometriosis, age-related reproductive decline, and both benign and malignant tumors, offering critical insights into their pathogenesis and identifying potential avenues for therapeutic intervention.

Key molecular players and pathways include the impact of autophagy, senescence, and proteolytic signaling on reproductive aging and fertility; the identification of critical regulators such as HIF-1α and NLRP3 in cancer and infertility; and the discovery of promising therapeutic targets such as BET proteins in uterine leiomyosarcoma and AURKA in ovarian cancer. Notably, recent advances in single-cell transcriptomics and spatial technologies have begun to redefine our cellular and molecular understanding of reproductive tissues, opening new opportunities for precision diagnostics and targeted therapies.

Collectively, the articles underscore the complexity and interconnectivity of biological systems that govern female reproductive health. They also reveal substantial knowledge gaps, particularly in the translation of basic discoveries to clinical practice, and underscore the importance of continued interdisciplinary research to develop effective, fertility-preserving treatments for women affected by these conditions.

By integrating current findings and exploring emerging directions, this Special Issue not only enhances our understanding of gynecological diseases but also serves as a catalyst for future studies aimed at improving the reproductive health and overall well-being of women worldwide.

## Data Availability

No new data were created or analyzed in this study. Data sharing is not applicable to this article.
